# Effects and mechanisms of the myocardial microenvironment on cardiomyocyte proliferation and regeneration

**DOI:** 10.3389/fcell.2024.1429020

**Published:** 2024-07-10

**Authors:** Kexiao Zheng, Yanglin Hao, Chenkun Xia, Shaoxian Cheng, Jizhang Yu, Zhang Chen, Yuan Li, Yuqing Niu, Shuan Ran, Song Wang, Weicong Ye, Zilong Luo, Xiaohan Li, Jiulu Zhao, Ran Li, Junjie Zong, Han Zhang, Longyong Lai, Pinyan Huang, Cheng Zhou, Jiahong Xia, Xi Zhang, Jie Wu

**Affiliations:** ^1^ Department of Cardiovascular Surgery, Union Hospital, Tongji Medical College, Huazhong University of Science and Technology, Wuhan, China; ^2^ Department of Anesthesiology, Union Hospital, Tongji Medical College, Huazhong University of Science and Technology, Wuhan, China; ^3^ Jingshan Union Hospital, Union Hospital, Tongji Medical College, Huazhong University of Science and Technology, Wuhan, China

**Keywords:** myocardial microenvironment, cardiomyocyte proliferation, cardiomyocyte regeneration, animal models, immunity, metabolism and cardiac dynamics

## Abstract

The adult mammalian cardiomyocyte has a limited capacity for self-renewal, which leads to the irreversible heart dysfunction and poses a significant threat to myocardial infarction patients. In the past decades, research efforts have been predominantly concentrated on the cardiomyocyte proliferation and heart regeneration. However, the heart is a complex organ that comprises not only cardiomyocytes but also numerous noncardiomyocyte cells, all playing integral roles in maintaining cardiac function. In addition, cardiomyocytes are exposed to a dynamically changing physical environment that includes oxygen saturation and mechanical forces. Recently, a growing number of studies on myocardial microenvironment in cardiomyocyte proliferation and heart regeneration is ongoing. In this review, we provide an overview of recent advances in myocardial microenvironment, which plays an important role in cardiomyocyte proliferation and heart regeneration.

## 1 Introduction

**FIGURE 1:**
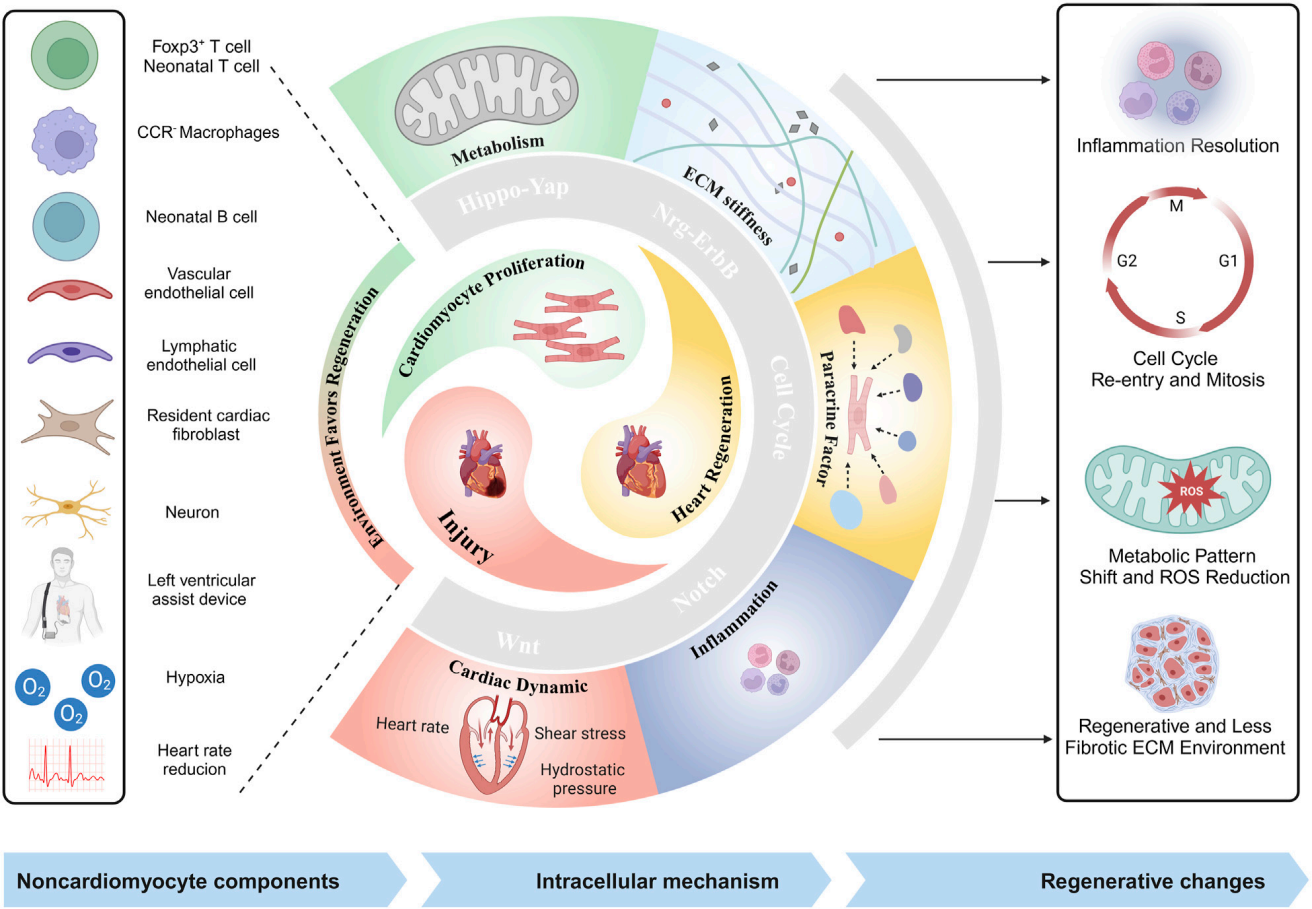
Illustrative schematic of noncardiomyocyte components, and mechanisms in heart regeneration (created with BioRender.com).

For a long time, it has been widely believed that the heart completes its growth and development prior to birth, subsequently transitioning into a still state that persists throughout life. The development of the heart involves dynamic alterations in its diverse cellular constituents. The process of cardiac development has three main stages: the formation of primitive myocardium, chamber formation, and the development of the heart’s electrical system (Katano et al., 2019). In humans, during the second embryonic week, after gastrulation, mesodermal cells that migrate anteriorly initiate heart development and acquire the potential to differentiate into cardiomyocyte lineage. (Buijtendijk et al., 2020). At this stage, mesodermal cells differentiate into primitive cardiomyocytes, exhibiting electrical activity and spontaneous contraction. In the third embryonic week, the ventricular chambers and the heart’s electrical conduction system develop alongside the cardiac chambers (Spurlock and Qian, 2023). By the eighth embryonic week, the heart completes its development and begins to integrate noncardiomyocyte cells (Flores et al., 2023). In mammals, the heart completes its development shortly after birth, and cardiomyocytes attain terminal differentiation (Tan and Lewandowski, 2020). Therefore, it is generally believed that the regenerative potential of the adult myocardium is restricted. Postnatal transition to a nonregenerative phase enables cardiomyocytes to sustain the heart’s demands and adapt to pulmonary circulation’s physiological challenges. However, an increasing number of studies have demonstrated that the human heart is not strictly postmitotic. Heart function of human newborn infants has been reported to be completely restored through regeneration after severe myocardial infarction (MI) (Haubner et al., 2016). Even when the heart structure and cardiomyocytes remain in a relatively quiescent state in adulthood, cardiomyocytes continue to undergo self-renewal at a very low rate (less than 1% per year) (Senyo et al., 2013). Increasing evidence suggests that the human heart indeed possesses a degree of regenerative capacity. This review aims to summarize the various microenvironments conducive to heart regeneration and their underlying mechanisms, in order to provide novel insights and directions for research in the field of cardiac regenerative medicine (Figure 1).

## 2 Heart regeneration in different species

Regeneration is a universal ability among organisms in nature. We can draw analogies from the heart regeneration processes observed in other classical regenerative species and explore how to achieve myocardial regeneration in mammals. It is generally believed that the regenerative capacity of a species is inversely proportional to its level of evolution. The more highly evolved a species or organ, the more limited is its regenerative capacity (Nguyen et al., 2021). Researchers have studied cardiomyocyte regeneration in many species (Table 1). Multiple studies show species-specific and developmental stage-dependent disparities in the regenerative capacity of cardiomyocytes. For instance, adult mammalian cardiomyocytes are stable, whereas zebrafish, a teleost, exhibit robust cardiomyocyte regeneration, serving as a leading model for heart regeneration studies to date (Vivien et al., 2016). In contrast, medaka fish, another teleost species, differs from zebrafish in its cardiac repair mechanism, featuring prominent fibrotic scarring and limited regeneration after injury (Ito et al., 2014). Among amphibians, salamanders can rapidly initiate the regeneration process and restore heart function after injury, whereas frogs lack this ability (Rumyantsev, 1973). The cardiomyocyte regeneration capacity of rodents varies among species and changes dramatically within a week after birth. Moreover, the processes of heart regeneration involve distinct cell types across species. It is interesting to look at these differences from a evolutionary perspective.

**TABLE 1:** Heart regeneration among zebrafish, amphibian and mammal.

Species	Teleost zebrafish	Urodele amphibian	Mammal
Cardiomyocyte	mononucleated, diploid	mononucleated, diploid	Neonates: diploid Adult: binuclear or polyploid
Heart structure	One atrium and ventricle	Two atria and a single ventricle Avascular ventricle	Two atria and ventricles
Cardiac regeneration rates	High	Moderate	Almost negligible (Limited to neonatal stage)
Regeneration process	Within seconds, a clot forms to stop bleeding. Hours later, inflammatory signals trigger endothelial cell changes, creating gaps filled by new cardiomyocytes. (Kikuchi et al., 2011)	First, a blood clot is rapidly formed, followed by the fibrin clot, and eventually the clot is gradually replaced by cardiomyocytes. (Witman et al., 2011)	Adult: blood clot is gradually replaced by scar tissue Neonates: The clot is gradually absorbed and replaced with new cardiomyocytes. (Porrello et al., 2011)
Speed of regeneration	30days (Kikuchi et al., 2010)	60–90 days (Witman et al., 2011)	Neonates 21day fully restored myocardium 2 months restored systolic function (Porrello et al., 2011)

### 2.1 Lower vertebrates: the teleost zebrafish and Urodelian amphibians

The teleost zebrafish, known for their remarkable regenerative abilities, is the most extensively studied model in heart regeneration research. In 2002, Kenneth et al. showed that the double-chambered heart of zebrafish can repair cardiac injury induced by a 20% ventricular resection and restore function through myocardial cell regeneration. Complete repair after injury through cardiomyocyte regeneration has been observed in various zebrafish heart injury models: apical resection injury, cryoinjury-induced cardiomyocyte ablation, genetic ablation, ischemia-reperfusion, laser-targeted ventricular ablation, and explant cultures (Parente et al., 2013). Studies on myocardial regeneration in adult zebrafish have demonstrated that the regenerated cardiomyocytes at the injury site consist primarily of preexisting cardiomyocytes that dedifferentiated after injury and re-entered the cell cycle under self-regulating conditions, subsequently migrating directionally to the injury site and differentiating into myocardial units with complete functional recovery (Jopling et al., 2010). Furthermore, the zebrafish heart’s regenerative capacity is not limited to cardiomyocytes alone. Vascular and endothelial cells also contribute to the healing process, ensuring adequate blood supply to the regenerating tissue (Ross Stewart et al., 2022). This natural regenerative capacity of the zebrafish heart provides valuable insights into the mechanisms underlying cardiac regeneration. Researchers are using the zebrafish model to identify genes, signaling pathways, and other factors that are essential for heart repair and regeneration (Zuppo and Tsang, 2020). Ultimately, the goal is to translate these findings from the zebrafish to human heart regeneration, with the hope of developing new therapeutic strategies for treating heart disease and injury in humans.

Despite anatomical differences, the adult zebrafish heart histologically resembles the precipitated embryonic mammalian heart, featuring a double-chambered, single-looped structure (Sorbini et al., 2023). As a nonmammalian model, zebrafish exhibit approximately 20% allelic overlap with mammals, making direct comparisons somewhat challenging (Chaoul et al., 2023). The functional differences in genes between zebrafish and humans are sufficient to disrupt any phenotypic similarities, and the different living environments of zebrafish and mammals also complicate the modeling of some functional studies (Lieschke and Currie, 2007). However, 70% of human protein-coding genes can be found in zebrafish, and 84% of disease-related genes have homologs in zebrafish, indicating that they can serve as important models for functional and disease research (Wynsberghe and Vanakker, 2023).

The study of urodele amphibians, particularly salamanders, has been a focus of regeneration research due to their ability to regenerate tissues, organs, and even body parts throughout life. As early as 1974, two studies demonstrated that the hearts of salamanders can undergo complete histological and functional regeneration after injury (Becker et al., 1974). After ventricular cardiomyocyte resection in salamanders, the injury site undergoes deposition of fibronectin similar to scar formation in mammals within the first 7 days; subsequently, there is an increase in the proliferation of epicardial cells and cardiomyocytes which gradually replace the deposited extracellular matrix (Piatkowski et al., 2013). Furthermore, these regenerating cardiomyocytes exhibit similar contractile function as their native counterparts, indicating a high degree of functional recovery (Piatkowski et al., 2013). Some studies have shown an increase in the expression of cardiac tissue markers such as Islet1 and Gata4 during the regeneration process, and other studies have shown that at least a portion of the myocardium undergoes dedifferentiation during regeneration (Witman et al., 2011). Unlike heart regeneration in zebrafish, this evidence suggests that the regenerated cardiomyocytes in salamanders are heterogeneous in origin and that different cardiomyocytes may have different regeneration sources. Furthermore, salamanders and zebrafish undergo distinct regeneration processes: the formation of extracellular matrix is bypassed during zebrafish regeneration, while conversely in salamanders the newly regenerated cardiomyocytes gradually replace the extracellular matrix trabeculae to complete regeneration.

### 2.2 Mammals

During the embryonic stage in mammals, cardiomyocytes express tissue-specific structural genes during proliferation (Bishop et al., 2022). Immediately after birth, chromatin remodeling occurs in cardiomyocytes, and mononuclear diploid cardiomyocytes become binuclear or polyploid (Rigaud et al., 2023). As a result, cardiomyocytes exit the cell cycle and continue to grow through cardiomyocyte hypertrophy, which is characterized by an increase in cell size alone (DUva et al., 2015). The energy production of cardiomyocytes relies primarily on glycolysis in the embryonic stage and gradually transitions to fatty acid oxidation metabolism postnatally to meet the energy requirements of adult cardiomyocytes (Menendez-Montes et al., 2023). Subsequent studies have demonstrated that thyroid hormone activation of the IGF-1/IGF-R/Akt pathway in binuclear cardiomyocytes during the first 15 days after birth triggers a brief but intense proliferation burst, leading to an increase in cardiomyocyte number of approximately 40% (Naqvi et al., 2014). In 2009, an experiment also demonstrated that human cardiomyocytes renew at a rate of 0.5%–2%, and this rate decreases with age (Alonaizan and Carr, 2022). However, compared to regeneration-capable animals such as salamanders and zebrafish, adult mammals do not initiate regenerative repair after injury and instead form permanent scar tissue. Under chronic pathological conditions (e.g., valve disease, hypertension, and postinfarction overload), adult cardiomyocytes typically reinitiate DNA synthesis without nuclear division, leading to further increases in nuclear ploidy (Laflamme and Murry, 2011).

Recently, regeneration after injury has been observed in neonatal mammals, similar to that observed in regeneration models (Nishiyama et al., 2022). In both the neonatal mammalian apical resection and left anterior descending coronary artery occlusion myocardial infarction models, cardiomyocyte regeneration has been shown to restore the damaged cardiac unit (Haubner et al., 2012). Subsequent series of injury models (such as ventricular resection, coronary artery ligation, and cryoinjury models) have further validated the robust regenerative capacity of cardiomyocytes in neonatal mice after injury (Castillo-Casas et al., 2023). The regenerative capacity of cardiomyocytes in neonatal mammals has been validated in numerous experimental models that simulate pathological loads, including models of aseptic inflammation of the myocardium, overload models of transverse aortic constriction, and unloading models of embryos with low hematocrit (Malek Mohammadi et al., 2019a). There is a consensus among researchers that neonatal mammals possess a narrow window for cardiomyocyte regeneration following birth. Within this window, cardiomyocytes demonstrate the capacity to restore heart function through regenerative repair following injury (Porrello et al., 2011). In mice, the regenerative capacity starts to decline at postnatal day 7 (P7), coinciding with the cardiomyocyte cell cycle arrest and binucleation (Bishop et al., 2021). The duration of this time window differs among mammals. For instance, in the porcine heart, this regenerative capacity appears to be limited to the first 2 days following birth (Ye et al., 2018).

## 3 Animal models used for heart regeneration research

**FIGURE 2:**
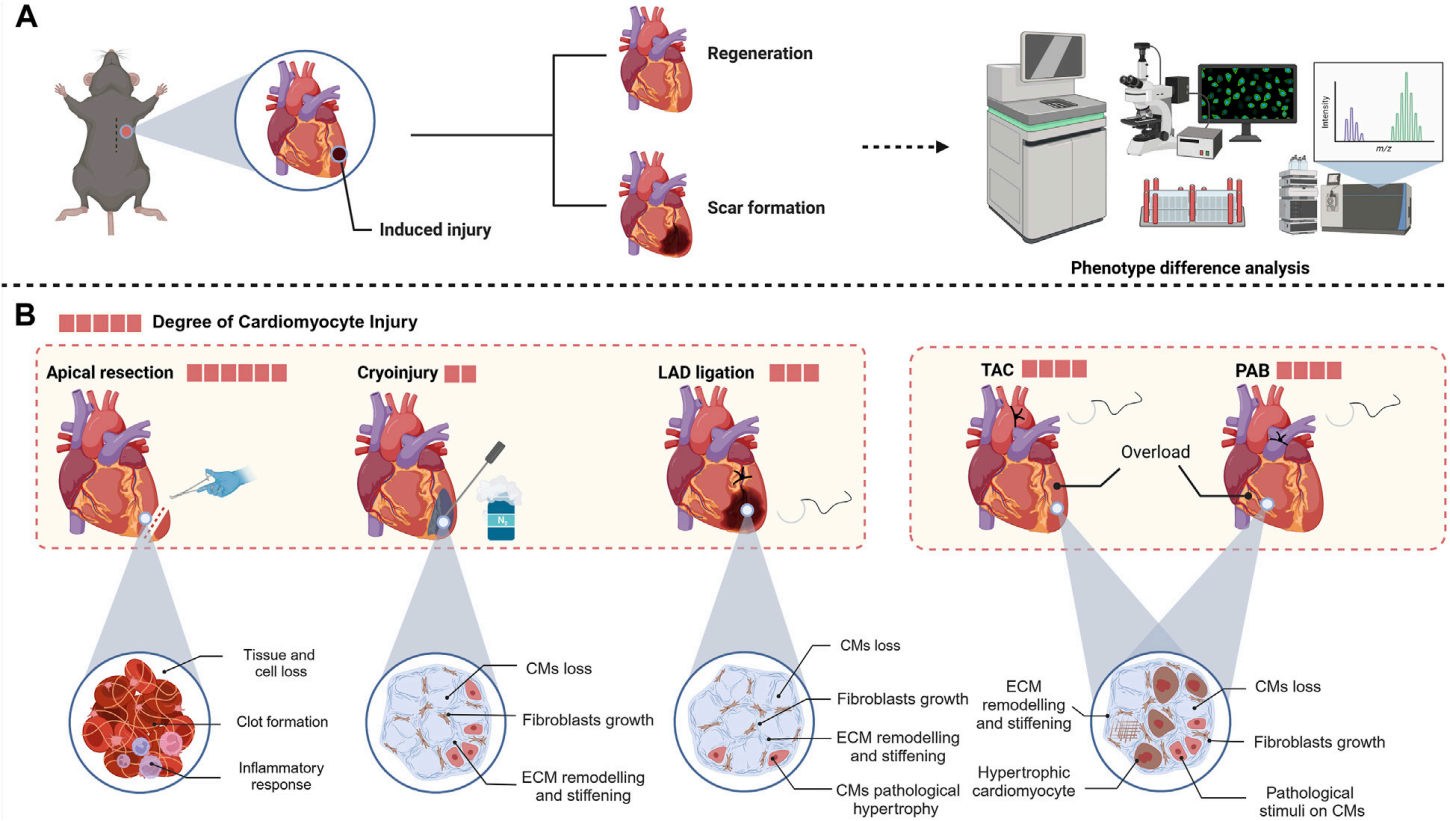
**(A)** Usual workflow for heart regeneration research. **(B)** Different animal models of heart injury, which induce varying degrees of damage to different heart components (created with BioRender.com).

After decades of research, the field of cardiac regeneration is gradually dispelling the long-held stereotype of “nonregenerative”. Currently, cardiac regeneration primarily encompasses two processes: first, the minimal self-renewal that occurs at a very low rate under physiological conditions, and second, the augmented regenerative capacity of the mammalian myocardium after injury (Bergmann et al., 2009). Research emphasis in the field of cardiac regeneration has shifted from merely demonstrating the regenerative capacity of cardiomyocytes to promoting meaningful repair of damaged hearts (Garbern and Lee, 2022). To meet this formidable challenge, researchers seek an effective and clinically relevant animal model of injury to better study cardiac regeneration. Currently, heart injury experimental models can be broadly categorized into two groups (Figure 2). The first category primarily encompasses nonischemic injuries, including the heart cryoinjury model, the apical resection model, and the second category of ischemic injury models (Sarig et al., 2019). These models directly result in cardiomyocyte loss, thereby facilitating the exploration of cardiomyocyte injury responses. Another category of injury is mainly induced by pressure overload models that lead to pathological remodeling and pathology of the ventricles, such as the transverse aortic constriction model and the pulmonary artery banding model (Malek Mohammadi et al., 2021).

### 3.1 Heart apical resection model

In 2011, Porrello et al. introduced the innovative application of apical resection to establish an injury model in neonatal mice (1 day postnatal) and observed complete recovery of the left ventricle within 21 days following a 10% resection, igniting significant enthusiasm in the field of cardiac regeneration (Porrello et al., 2011). Apical resection surgery not only directly causes the loss of cardiomyocytes but also leads to significant loss of non-cardiomyocytes. When constructing animal models, it is necessary to perform open-chest surgery under deep anesthesia at low temperatures and quickly remove the minimum amount of tissue from the left ventricular chamber (Fgure2) (Mahmoud et al., 2014). After apical resection, the immediate inflammatory response and clot formation seal the injury site (Tarnavski et al., 2004). The high mortality rate associated with this method necessitates meticulous aseptic conditions and surgical expertise. Typically, apical resection removes approximately 10%–20% of healthy ventricular tissue (Xiong and Hou, 2016). The apical resection surgery is usually performed only on one to seven days postnatal mice, as the ability of these mice to form blood clots to seal the surgical wounds diminishes with age, leading to increased mortality rates (Mahmoud et al., 2014). One study has questioned the phenomenon of regeneration induced by apical resection in neonatal mice. Andersen et al. performed apical resection or sham surgery on hundreds of mice, but only extensive scar tissue formation was observed in the hearts of the apical resection group at 21 days post-surgery (Andersen et al., 2014). The reasons for the differences between Porrello and Andersen are not fully understood, but it appears that the apical resection model in neonatal mice does indeed lead to regeneration, as more laboratories have since conducted research on this model (Sadek et al., 2014). Many hypotheses suggest that numerous intermediary factors exist between apical surgery and myocardial regeneration, potentially impacting the observed outcomes.

### 3.2 Heart cryoinjury model

In the cryoinjury model, researchers use a cryoprobe cooled with liquid nitrogen to precisely damage the ventricular muscle (Figure 2). This injury causes the death of cardiomyocytes in the affected region, subsequently initiating the process of scar formation, which closely mimics the alterations in the functional units of the heart that occur following MI in humans (Polizzotti et al., 2016). In 2011, Chablais et al. first used this model to create a local injury of approximately 25% in the ventricular myocardium of zebrafish (Chablais et al., 2011). In Chablais’s study, cryoinjury caused a significant loss of cells in the affected area, with necrotic cells being cleared away and replaced by extracellular matrix. Subsequently, myocardial tissue regeneration occurred, gradually replacing the extracellular matrix that had accumulated postinjury, ultimately leading to the complete restoration of the damaged region (Chablais et al., 2011). In Hein et al.'s study, there was a delay in ventricular regeneration following cryoinjury, with complete recovery of heart function taking up to 180 days, significantly longer than the 45 days required for a heart apical resection model (Hein et al., 2015).

To better investigate whether a zebrafish-like myocardial regeneration program also exists in mammals, the induction of cardiomyocyte ablation using cryoprobes has also been applied in mammalian myocardial injury models (Strungs et al., 2013). Darehzereshki et al. have pioneered the development of models of varying degrees of cryoinjury to induce transmural or nontransmural infarction, observing the impact of the severity of injury on regeneration (Darehzereshki et al., 2015). In their experiments, the hearts of mice with nontransmural injuries demonstrated recovery of cardiac function, whereas mice with transmural injuries exhibited signs of scar formation and no signs of regeneration or recovery of function up to 120 days post-injury71. The necrotic process caused by cryoinjury prevents this model from fully simulating the ischemic injury or representing the pathological process of human MI well. However, in comparison to the apical resection model, the cryoinjury model exhibits reduced cardiac damage and can induce larger cardiomyocyte defects. Additionally, because cardiac fibroblasts and collagen are resistant to cryoinjury, the low temperature mainly damages cardiomyocytes, allowing researchers to better investigate the regeneration mechanism involved in cardiomyocyte injury (Cox et al., 2021).

### 3.3 Left anterior descending coronary artery ligation model

The LAD ligation murine model, a frequently utilized approach for studying MI, accurately simulates the postinfarction pathological processes through the use of permanent ligature to obstruct blood flow to the myocardium via the LAD (Castillo-Casas et al., 2023). Once the heart is exposed, occlusion of the LAD effectively prevents blood flow to the affected area, while the surrounding myocardial tissue remains largely unaffected (Fischer et al., 2022). Using the LAD coronary artery ligation model, numerous researchers have consistently demonstrated the remarkable regenerative capacity of neonatal mouse cardiomyocytes following ischemic injury (Liu et al., 2021). However, in the P1 LAD ligation model, Konfino et al. observed incomplete regeneration accompanied by small scars at 28 days, with some mice also developing aneurysms (Figure 2) (Konfino et al., 2015).

In other studies, researchers have conducted experiments in which the heart was ligated and inserted into the chest, with one end of the ligature remaining outside the chest. Once ischemic changes were observed via electrocardiogram or after 5 minutes, the ligature was withdrawn to restore the blood supply to the cardiomyocytes. This ischemia-injury model is similar to the pathological processes observed in patients who underwent stent placement following coronary occlusion (Li X. et al., 2023). Using this model, Li Xiang et al. reported that adult mouse cardiomyocytes with knockout of fatty acid oxidation genes can re-enter the cell cycle after ischemia-reperfusion injury, completely preventing scar formation (Li X. et al., 2023).

### 3.4 The pressure overload model of the heart with aortic constriction

In addition to factors such as MI that cause the loss of cardiomyocytes, pressure overload is a frequent cause of cardiac stress. In diseases such as congenital cardiomyopathy and valvular heart disease, pathological pressure overload not only causes chronic loss of cardiomyocytes but also leads to pathological remodeling of the ventricles, which in later stages can increase interstitial fibrosis and capillary rarefaction (Bassat et al., 2017). The pressure overload model of the heart with aortic constriction is an animal model in which afterload on the heart is increased by narrowing the aorta, resulting in pressure overload of the heart (Figure 2).

Pulmonary artery banding (PAB) is a palliative surgery used to prevent pulmonary hypertension, but it can cause excessive overload of the right ventricle. It has been used in some experiments to construct a neonatal mouse model of right ventricular hypertrophy, simulating certain pathological conditions(Figure 2) (Wang et al., 2017). In 2020, Ye et al. induced pressure overload in P1 mice using pulmonary artery banding (PAB); through RNA sequencing technology, they found that this model can lead to neonatal heart proliferation and prolong the postnatal proliferation window of cardiomyocytes by more than 7 days (Ye et al., 2020). This method simulates the pathological conditions associated with congenital heart diseases that frequently result in right ventricular pressure overload, including Tetralogy of Fallot, pulmonary hypertension, and pulmonary stenosis (Vriz et al., 2020).

Similarly, a left ventricular overload animal model can be constructed by narrowing the aorta to induce high pressure in the left ventricle. Mohammadi et al. performed transverse aortic constriction (nTAC) surgery in neonates at P1 and P7 and reported that pathological outcomes were prevented in P1 mice following it (Malek Mohammadi et al., 2019b). This model can also be applied to the construction of animal models of pathological myocardial remodeling. Mortimer et al. constructed a pressure overload model using TAC surgery and demonstrated the protective effect of bone marrow-derived growth factors on cardiomyocytes under overload pressure (Korf-Klingebiel et al., 2021).

## 4 The extracellular microenvironment of cardiomyocytes favors heart regeneration

Cardiomyocytes constitute approximately 75% of the total volume of the adult mammalian heart but only approximately 25%–35% of its total number of cells (Trager et al., 2023). The proportion of nonparenchymal cells in the heart exceeds the number of cardiomyocytes; approximately half of the cells are fibroblasts, and one-fourth are endothelial cells (EC) (Litviňuková et al., 2020). The functional status of the heart is jointly determined by the complex interplay between cardiomyocytes and other cell types. Most previous research on myocardial regeneration has been centered solely on cardiomyocytes. However, with advancements in sequencing technology and spatial transcriptomics, a growing number of myocardial regeneration phenomena have come to light, thereby highlighting the significance of the myocardial microenvironment’s impact on the regeneration of cardiomyocytes. (Table 2) (Wu et al., 2020). In this section, we aim to characterize the cardiac microenvironment and the dynamic interactions between cells during tissue injury and regeneration.

**TABLE 2:** Modulators from noncardiomyocyte cells that promote CM proliferation.

Non-CM cell	Signal	Mechanism	Function	Ref
Macrophage	Mydgf	c-Myc/FoxM1 pathway	Induce cardiomyocyte proliferation in adult mice after MI	Wang et al. (2020a)
	OSM	Gp130/Src-YAP pathway	Promote cardiomyocyte proliferation and heart regeneration in adult mice after MI	Li et al. (2020c)
CCR2^−^ macrophages	Less pro-inflammatory cytokines	Inflammation resolution and angiogenesis	Cardiac repair and regeneration in neonatal mice after MI	Molinaro et al. (2023)
Foxp3^+^ T cell	Anti-inflammation signal	Activation of repair-promoting macrophages	Cardiac repair and regeneration	Weirather et al. (2014)
	CST7, TNFSF11, IL33, FGL2, MATN2, IGF2	Paracrine pro-proliferative factors	Promote fetal and maternal CM proliferation	Zacchigna et al. (2018)
	CCL24, GAS6, AREG	Paracrine factors	Potentiate neonatal cardiomyocyte proliferation	Li et al. (2019)
T cell	PD-1	PD-1–PD-L1 pathway	Prevent excessive and prolonged inflammation in neonatal mice	Michel et al. (2022)
B cell	Paracrine proteins	Inhibit inflammatory responses, promote angiogenesis and the clearance of cellular debris	B cell depletion did not exhibit regeneration after apical resection in neonatal mice Insufficient to offset the anti-regenerative effects in adult mice	Tan et al. (2023)
LEC	REELIN Tbx1	ERK/Akt pathway Activation integrin-β1 CCL2, ICAM 1	Promote CM proliferation and heart rapier in neonatal mice after MI Promotes an immunosuppressive microenvironment for CMs regeneration	Liu et al. (2020)
				Wang et al. (2023d)
	REELIN	Rac1-Yap pathway	Promote cardiomyocyte proliferation	Pei et al. (2023)
VEC	LPA	LPA-Lpar2 pathway	Promote neonatal heart regeneration	Pei et al. (2022)
Cardiac EC	circWhsc1	TRIM59/STAT3/Cyclin B2 pathway	Promote cardiomyocyte proliferation and heart regeneration in adult mice after MI	Wei et al. (2023a)
	Mydgf	c-Myc/FoxM1 pathway	Induce cardiomyocyte proliferation in adult mice after MI	Wang et al. (2020a)
Vagal nerve	Nrg1, Ngf	Mediate inflammation	Inhibition reduces cardiomyocyte proliferation in the injured hearts of both zebrafish and neonatal mice	Mahmoud et al. (2015)
Sympathetic neuron	SN signal	*Per1/Per2*-wee1 kinase pathway	Downregulation of clock gene Per1/Per2 accompanied by upregulation of cell cycle genes	Tampakakis et al. (2021)
FB/ECM	Agrin	dystrophin-glycoprotein complex Yap/ERK pathway	Promote cardiac regeneration in neonatal adult mice after MI	Bassat et al. (2017)
	Versican	integrin-β1, ERK 1/2 and Akt downstream	Promote cardiac regeneration in neonatal adult mice after MI	Feng et al. (2023)
	Periostin	PI3K/GSK3β/CyclinD1 signaling pathway	Ablation of periostin suppresses post-infarction myocardial regeneration in neonatal mice	Chen et al. (2017)

### 4.1 Immune cells

Immune cells infiltrate the heart at gestation and remain in the myocardium, where they participate in the housekeeping functions of maintaining environmental homeostasis throughout life. The heart of a healthy adult mouse contains all major leukocyte classes, with a frequency 12 times greater than in skeletal muscle (Ramos et al., 2017). Macrophages are ontogenically diverse, heterogeneous, and the most abundant immune cells; they are interwoven within the cardiac parenchyma, and account for approximately 7% of the noncardiomyocytes (Thorp, 2023). Heart macrophages are composed of multiple subpopulations with divergent origins, including long-lived yolk sac-derived macrophages and adult monocyte-derived macrophages (Wang Z. et al., 2023). A study showed that the macrophages from circulating blood account for a small proportion of the macrophages in the heart (Bajpai et al., 2018). The macrophages in the heart mostly originate from fetal liver monocyte progenitors or are produced at birth (Wang Z. et al., 2020). High-resolution imaging of the mouse heart has revealed that cardiac-resident macrophages are ubiquitously distributed, at approximately 3×10^5^ per heart, and are highly dense around the ventricular myocardium with each cardiomyocyte surrounded by five heart-resident macrophages (Nicolás-Ávila et al., 2020). Cardiac-resident macrophages are identified as CD45^+^ CD11b^+^ F4/80^+^ and can be divided into at least four subsets based on the expression of MHC class II and Ly6C (Epelman et al., 2015). Moreover, there are cardiac-resident mast cells and dendritic cells (Hermans et al., 2019). Approximately 1% of the total number of cardiac leukocytes are resident cardiac dendritic cells (DCs), and the aortic valve is particularly rich in antigen-presenting cells (Choi et al., 2009). In addition to these resident cells, the heart also contains monocytes, neutrophils, B cells, and T cells, which mainly originate from circulating blood (Embryonic and Adult-Derived Resident Cardiac Macrophages Are Maintained through Distinct Mechanisms at Steady State and during Inflammation, 2014).

#### 4.1.1 Macrophages

Macrophages are the most abundant type of immune cell in the normal heart and in the injured heart after MI (Sager et al., 2016). There are at least four transcriptionally distinct cardiac macrophage subsets in the healthy murine myocardium, and this number rises after MI (Dick et al., 2019). Macrophages are crucial in tissue regeneration and repair and mediate biphasic responses of myocardial injury and repair. Research has demonstrated macrophages’ indispensable role in myocardial regeneration by showing that neonates depleted of macrophages are unable to regenerate cardiomyocytes and form fibrotic scars (Aurora et al., 2014). Among macrophages, two residential subpopulations (hbaa^+^ cardiac-resident macrophages and timp4.3^+^ cardiac-resident macrophages) were enriched only in regenerative zebrafish hearts (Wei K.-H. et al., 2023).

Although cardiac regeneration occurs only in a narrow developmental window in mammals, identifying this window’s characteristics in terms of resident macrophages, in addition to intracellular factors, will also be particularly relevant. A recent study showed that the transcriptional profile, magnitude, and polarization of the monocyte/macrophage response differed between neonatal mice younger than 7 days (regenerative) and neonatal mice older than 7 days (nonregenerative) (Aurora et al., 2014). Distinct macrophage subsets contribute to disparate patterns of cardiac regeneration and remodeling in the neonatal and adult heart (Zuo et al., 2023). There is only one CCR2^−^/MHC-II^low^ subset of embryonic-derived macrophages and one CCR2^+^ subset of monocytes in the neonatal mouse heart (Li J. et al., 2023). In response to injury neonatal mice selectively expand the CCR2^−^/MHC-II^low^ subset macrophages without recruiting additional CCR2+ subset monocytes (Lavine et al., 2014). These CCR2^−^ macrophages isolated from the injured neonatal mouse hearts release lower levels of proinflammatory cytokines (Lavine et al., 2014). In adult hearts, the CCR2^−^/MHC-II^low^ subset is replaced by proinflammatory monocytes and monocyte-derived macrophages expressing CCR2^+^/MHC-II^high^, which have limited ability to promote reparative activities and generate inflammation or oxidative stress instead (Lavine et al., 2014).

**FIGURE 3:**
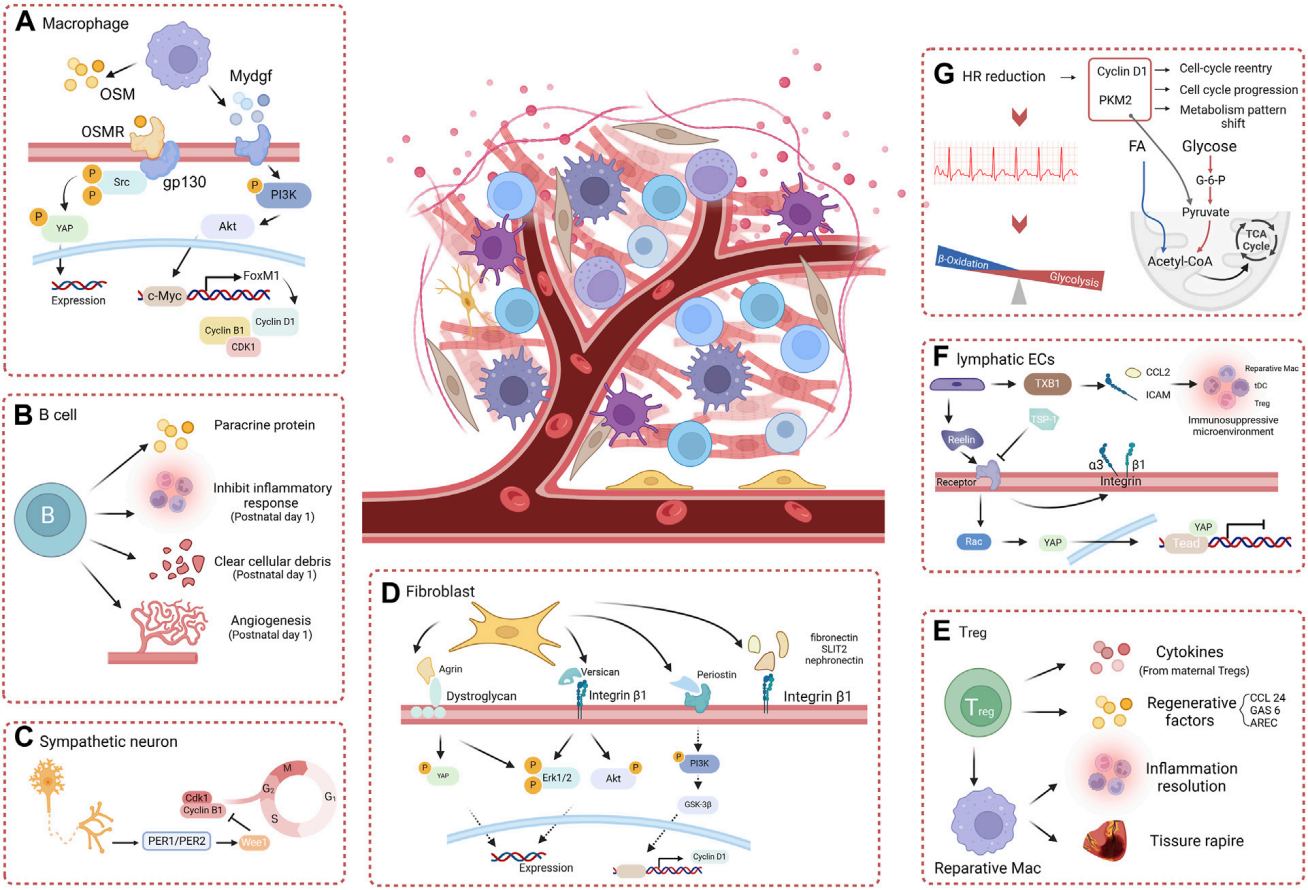
The role of myocardial microenvironment in heart regeneration. **(A)** Macrophage paracrine factors effectively promote heart regeneration through the OSM-gp130 pathway and the Mydgf-PI3K-Akt pathway. **(B)** B cells effectively promote heart regeneration by directly secreting paracrine factors and regulating non-cardiomyocytes. **(C)** Sympathetic neurons regulate the cardiomyocyte cell-cycle and heart regeneration through the PER1/PER2-Wee1 pathway. **(D)** The extracellular matrix components Agrin, Versican, and periostin, via integrins or dystroglycan, stimulate cardiomyocytes proliferation and heart regeneration. **(E)** Treg cells direct paracrine factors or promote reparative macrophages to enhance cardiomyocyte proliferation and heart regeneration. **(F)** Lymphatic ECs directly stimulate cardiomyocyte proliferation through Reelin and interact with other noncardiomyocyte cells via TXB1 to create a favorable environment for heart regeneration. **(G)** Heart rate reduction effectively promotes a shift in the metabolic pattern of cardiomyocytes and stimulates the mitotic process, enhancing cardiomyocyte proliferation and heart regeneration (created with BioRender.com).

Cardiac resident macrophages crosstalk with other noncardiomyocytes and serve as key regulators in stimulating angiogenesis and inhibiting fibrosis to promote regeneration after injury. A 2020 study by Li et al. showed that mechanistically, macrophages that infiltrate the injured myocardium in neonatal mice secrete oncostatin M (OSM, a pleiotropic secretory protein belonging to the IL-6 family) (Li Y. et al., 2020). OSM binds to the OSMR/gp130 receptor in cardiomyocytes and activates the Src-YAP signaling pathway, thereby inducing myocardial proliferation and cardiac regeneration(Figure 3A) (Li Y. et al., 2020). Remarkably, in the infarcted cardiomyocytes of adult mice, conditional activation of gp130 is sufficient to initiate cardiac regeneration through Src-YAP-mediated CM proliferation (Li Y. et al., 2020). A study showed that myeloid-derived growth factor (Mydgf), a paracrine protein mainly secreted by bone marrow-derived macrophages, can effectively stimulate CM proliferation through the c-Myc/FoxM1 pathway (Korf-Klingebiel et al., 2015; Wang Y. et al., 2020). However, it is worth noting that in injured neonatal hearts, MYDGF is primarily derived from ECs(Figure 3A) (Wang Y. et al., 2020).

#### 4.1.2 T cells

Cardiac T cells accumulate mainly because of recruitment from the circulating T-cell pool, whereas local proliferation also contributes to their expansion. Although T cells are not the most abundant leucocyte subset in the heart, activated T cells can express many proinflammatory cytokines, which impact the activity of all other myocardial subsets. Thymic T-cell development typically occurs in two discontinuous periods: initial development on embryonic days 13–18, and a second wave of development on postnatal days 3–6 (Lavaert et al., 2020). After activation, circulating CD4^+^ T cells are mobilized to the damaged heart region, where they promote the activity of CCR2+ macrophages to participate in the myocardial injury response (Yan et al., 2013). According to their phenotypes, cytokine expression profiles, and functions, CD4^+^ T cells can be further divided into T helper type 1 (Th1), T helper type 2 (Th2), and regulatory T-cell (Treg) subsets (Hofmann et al., 2012). The mechanism of T-cell stimulated cardiac regeneration response is currently unclear, and it is likely to be multifactorial, involving communication between several cell types (USSCs, immune cells, T cells, and hypoxic cardiomyocytes), as well as paracrine regenerative factors (Hu et al., 2023).

The Foxp3^+^ Treg subset is activated in the later stage of injury and contributes to inflammation resolution and tissue repair through the activation of repair-promoting macrophages (Xia et al., 2020). During pregnancy, Tregs expand in the mother, resulting in maternal immune tolerance to the fetus (Aluvihare et al., 2004). Study has shown that Tregs can promote fetal heart development and maternal myocardial proliferation during pregnancy and even exert an immune-independent regenerative effect on MI in adulthood (Zacchigna et al., 2018). Although there is no direct evidence that maternal Tregs can traverse the placenta, they have been detected in fetal lymph nodes, and six cytokines (Cst7, Tnfsf11, Il33, Fgl2, Matn2, and Igf2) secreted by maternal Tregs within the fetal heart have the capacity to stimulate cardiomyocyte proliferation individually(Figure 3E) (Zacchigna et al., 2018). Therefore, the separation of maternal blood supply during childbirth may also be one of the reasons for the decrease in regenerative capacity during the postnatal window for heart regeneration in neonatal mammals. A study showed that in neonatal NOD/SCID mice deficient in T cells, resident macrophages alone failed to induce cardiac regeneration, causing more severe fibrosis instead, but that adoptive transfection of Foxp3^+^ Tregs restored cardiac regeneration (Li et al., 2019). Single-cell transcriptomic profiling revealed that Tregs directly potentiate CM proliferation through the paracrine regenerative factors CCL 24, GAS 6, and AREC (Li et al., 2019). Direct cell-cell contact between T cells and small cTnT^+^ cells forms during the early stages of regeneration and may facilitate the direct exchange of soluble factors (Ding et al., 2023).

Other studies found that CD4^+^ Th1 (TNF-α and IFN-γ) and Th17 (IL-17A) cells can directly inhibit the proliferation and promote the apoptosis of neonatal cardiomyocytes through related cytokines(Figure 3E) (Li J. et al., 2020). Consistent with previous studies, adult mice exhibit more effective CD4^+^ Th1 and Th17 responses than neonatal mice, and Th17 cells that produce IL-17 are less abundant in neonatal mice (Hofstetter et al., 2007).

#### 4.1.3 B cells

As a type of prominent acquired immune cell, B cells are believed to display a proinflammatory effect and exacerbate myocardial damage. Other studies have also indicated that adult cardiac B cells can promote CM proliferation in adult mice. (1) IL-10-producing B cells abundant in adult murine pericardial adipose tissue can terminate MI-induced inflammation to facilitate tissue repair/regeneration (Wu et al., 2019). (2) Bone marrow-derived naive B lymphocytes were found to improve cardiac function after MI in adult mice (Xu et al., 2022). Certain distinct subsets of B cells can also play a beneficial role in the repair of cardiac injury, promoting cardiomyocyte regeneration via paracrine proteins, although this effect is insufficient to offset proinflammatory and profibrotic effects (Tan et al., 2023). Single-cell RNA sequencing showed that cardiac B cells in postnatal day 1 mice exhibit increased abilities to inhibit inflammatory responses and promote angiogenesis and the clearance of cellular debris after myocardial injury(Figure 3B) (Tan et al., 2023). Neonatal mice with B-cell depletion did not exhibit any signs of regeneration after apical resection; this sheds new light on the indispensable roles of neonatal B-cell subsets in heart regeneration(Figure 3B) (Tan et al., 2023).

### 4.2 Cardiac fibroblasts and extracellular matrix

Fibroblasts, which constitute approximately 20% of the noncardiomyocytes, are the most numerous cell type in the heart and play crucial roles in heart development, homeostasis, and disease. Cardiomyocytes are embedded within a rich ECM, a locally secreted macromolecular network whose components include collagen, glycoprotein, proteoglycans, and glycosaminoglycan. The complex network of the extracellular matrix synthesized by fibroblasts with embedded cellular components forms the structure of the heart (Belviso et al., 2022). The myocardial extracellular matrix is a dynamically changing network of diverse components with various proteins having growth factor and cell receptor-binding properties (Rienks et al., 2014). Pathological changes in the adult heart lead to extensive remodeling of the extracellular matrix, ultimately resulting in scar formation and a decrease in heart function. As the heart develops during embryogenesis, the number of resident fibroblasts increases and these cells continue to expand postnatally on day 4 after birth, which is consistent with the initial formation of binucleated cardiomyocytes (Ivey et al., 2018). The extracellular matrix may also be involved in changes in the postnatal window for heart regeneration in neonatal mammals. In a coculture system, embryonic cardiac fibroblasts consistently induced proliferation of cardiomyocytes through β1-integrin paracrine signaling, in contrast to adult cardiac fibroblasts, which promoted myocyte hypertrophy (Ieda et al., 2009).

Previous studies have shown that changes in the extracellular matrix composition significantly impact CM growth and differentiation. In the zebrafish heart, regeneration is associated with sharp increases in specific ECM proteins and with an overall decrease in collagens and cytoskeletal proteins (Garcia-Puig et al., 2019). A study conducted by Chen et al. showed that ablation of periostin, an extracellular matrix protein, inhibits myocardial regeneration in neonatal mice after injury (Chen et al., 2017). The application of the GSK3β inhibitor SB216763 reversed this effect, indicating that this effect was modulated by the PI3K/GSK3β/CyclinD1 signaling pathway(Figure 3D) (Chen et al., 2017). Agrin is an extracellular matrix protein that has been widely studied for its involvement in the formation of neural synapses. Bassat et al. showed that recombinant agrin protein promotes the differentiation of induced pluripotent stem cells from humans and mice into CMs *in vitro* through the disassembly of the dystrophin-glycoprotein complex and YAP/ERK signaling pathway(Figure 3D) (Bassat et al., 2017). A single administration of agrin *in vivo* can promote cardiomyocyte regeneration after MI in adult mouse cardiomyocytes (Bassat et al., 2017). Recent studies showed that conditional knockout of Vcan in cardiac fibroblasts reduced cardiomyocyte proliferation and impaired neonatal heart regeneration (Feng et al., 2023). It has also been shown that versican, a chondroitin sulfate proteoglycan, can activate integrin β1 and downstream signaling molecules, including ERK 1/2 and Akt, thereby enhancing CM proliferation and promoting heart repair(Figure 3D) (Feng et al., 2023). Other ECM components, such as fibronectin, SLIT2, and nephronectin, also influence CM proliferation via integrin β1, indicating a crucial role for integrin β1 in bridging ECM signaling with CM proliferation (Wu et al., 2020).

### 4.3 Endothelial cells (ECs)

Endothelial cells (ECs) line the lumen of the heart, blood vessels, and lymphatic vessels, regulating blood flow, vascular plasticity, and the inflammatory response. Classes of cardiac ECs include endocardial ECs, vascular ECs, and lymphatic ECs. Quiescent ECs account for approximately 24% of cardiac homeostasis and are continuously monitored by the cardiovascular system to ensure their proper cardiac function (Litviňuková et al., 2024). During embryonic heart development, veins are the primary source of ECs, which support the growth of coronary vessels that facilitate the expansion of cardiomyocytes (Klaourakis et al., 2021). However, in adulthood, it is challenging for endocardial cells to acquire the characteristics of vascular ECs after injury, thus limiting the regenerative capacity of the adult heart (Trimm and Red-Horse, 2023). Restoration of functional blood and lymphatic vascular networks in the infarct and border regions via neovascularization and lymph angiogenesis is a key requirement for facilitating myocardial regeneration. In this process, ECs can also release growth factors and inflammatory cytokines and interact with other cells to promote the regeneration and repair of cardiomyocytes. In one study, the cardiac endothelial Brg1-Kdm7aa-Notch axis was demonstrated to regulate myocardial proliferation and regeneration via modulating the H3K4me3 at notch promoters in zebrafish (Xiao et al., 2023).

A study in 2015 demonstrated the heterogeneous origin of the cardiac lymphatic network independent of sprouting from veins: lymphatic progenitor cell populations derived from the yolk sac hemogenic endothelium can significantly regenerate after injury by treatment with VEGF-C in the adult heart (Klotz et al., 2015). In regenerating adult zebrafish hearts, the cryoinjury leads to extensive lymphangiogenesis of lymphatic vasculature. Blocking the Vegfc/Vegfr3 signaling inhibits the development of intact cardiac lymphatic vessels and increases the scar size of heart (Harrison et al., 2019). These studies have demonstrated the indispensable role of EC signaling in myocardial regeneration. In the neonatal mouse heart, the expression of RELN, the lymphoangiocrine signal regulating heart growth, steadily decreases from P2 to P14, coinciding with the loss of cardiac regenerative potential(Figure 3F) (Lymphoangiocrine signals promote cardiac growth and repair | Nature, n. d.). Liu et al. also observed that the conditioned medium derived from lymphatic ECs can promote the proliferation of cardiomyocytes cocultured by activating the ERK/Akt signaling pathway (Parente et al., 2013). They demonstrated that Reelin secreted by lymphatic ECs activates integrin-β1, leading to the regulation of cardiomyocyte proliferation and participation in regenerative repair after injury in neonatal mice (Lymphoangiocrine signals promote cardiac growth and repair | Nature, n. d.). Wang et al. showed that after MI, the transcription factor Tbx1, which attracts tDCs, Tregs, and reparative macrophages via chemokine Ccl21 and integrin Icam1, is upregulated in cardiac lymphatic ECs, promoting an immunosuppressive microenvironment for cardiomyocyte regeneration (Wang W. et al., 2023). Recently, Wei et al. reported that the Reelin and TSP-1 signals, which have opposing effects on CM proliferation, simultaneously act on the VLDLR receptor on CMs, with Rac1 and YAP serving as downstream (Pei et al., 2023). The restoration of blood supply following injury is a pivotal process for cardiac regeneration (Mathison and Rosengart, 2018). Recent studies have shown that, during angiogenesis after MI, vascular ECs can also promote heart regeneration in neonatal and adult mice, independent of blood supply recovery. During artery reassembly, arterial ECs, which migrate from arteries and reassemble into collateral arteries in neonates, have special reparative properties and are required for neonatal heart regeneration (Das et al., 2019). Pei et al. demonstrated that the LPA-LPAR2 signal pathway, which is required for neonatal heart regeneration, is activated after MI in vascular endothelium(Figure 3F) (Pei et al., 2022). In summary, endothelial cells play an important role in myocardial regeneration, including promotion of the formation of new blood vessels, regulation of the immune response, and cardiomyocyte protection. Further investigation into the mechanisms of ECs in myocardial regeneration is expected to provide new ideas and methods for the prevention and treatment of cardiovascular diseases.

### 4.4 Neuronal cells

The heart is governed by the extrinsic sympathetic and parasympathetic nervous systems and is influenced by the intrinsic cardiac nervous system, which is situated within its different chambers (Zhao et al., 2023). Although neuronal cells constitute less than 2.5% of human cardiac cells, they can regulate almost all physiological functions of the heart—including chronotropy, dromotropy, lusitropy, and inotropy—via the autonomic nervous system, which comprises both a sympathetic and a parasympathetic branch (Fukuda et al., 2015). One study has revealed that the neonatal regenerative heart undergoes a distinctive process of physiological reinnervation, which is contingent upon collateral artery formation (Salamon et al., 2023). Mahmoud et al. reported that chemical sympathectomy and vagal nerve resection, despite their opposite physiological effects, can significantly regulate heart regeneration after injury in postnatal mice (Mahmoud et al., 2015). In the same study, transcriptional profiling following vagal nerve resection revealed a blunted expression of inflammatory genes, a potential mechanism by which the autonomic nervous system can regulate cardiac regeneration by modulating immune function (Mahmoud et al., 2015). Recently, Tampakakis et al. presented evidence that the inhibition of sympathetic innervation *in vivo* leads to an increase in cardiomyocyte count, which is associated with the downregulation of the clock genes Period1 and Period2 and the concurrent upregulation of cell cycle genes(Figure 3C) (Tampakakis et al., 2021).

## 5 Oxygen and metabolism

**FIGURE 4:**
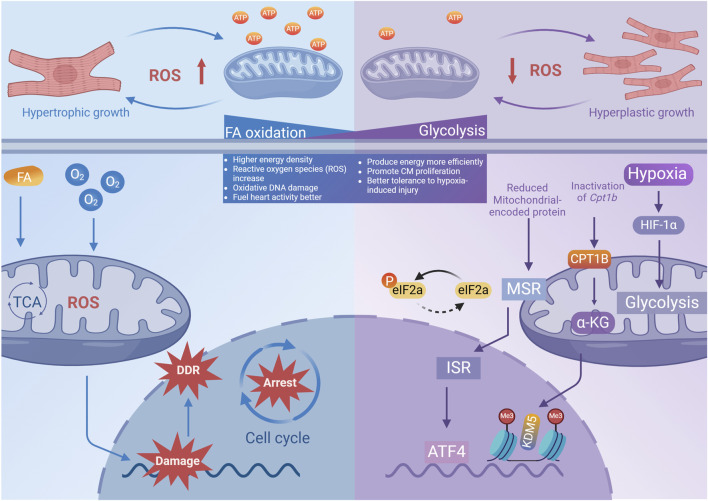
The impact of different metabolic patterns on cardiomyocyte proliferation. Metabolic pattern shift from FA oxidation to Glysis enhances the regenerative abilities of cardiomyocytes and the heart’s ability to withstand damage, by efficiently harnessing energy and reducing mitochondrial ROS production (created with BioRender.com).

As a result of the heart’s constant diastole (relaxation) and systole (contraction), cardiomyocytes are under significant metabolic stress. A significant circulatory difference before and after delivery in mammals is the change in oxygen supply (Fisher et al., 1980). In the embryonic stage of mammals, the arteriovenous duct is not necessarily present, and there is significant mixing of arterial and venous blood (Lopaschuk and Jaswal, 2010). Birth is accompanied by a shunt of arterial and venous blood, which greatly changes the oxygenation status of the heart. As cardiomyocytes mature during the development process, energy substrates shift from glycolysis to fatty acid oxidation to meet the myocardial metabolic energy requirements(Figure 4) (Li M. et al., 2023). Puente et al. suggested that the postnatal switch in energy metabolism toward the mitochondria results in more efficient production of energy, but also leads to more electron leakage in the electron transport chain, which generates mitochondrial oxygen radicals and activates the DNA damage response, inducing cardiomyocyte arrest (Puente et al., 2014). Gao et al. demonstrated that knockdown of mitochondrial protein translation directly triggers mitochondrial stress response (MSR) and integrated stress response (ISR), resulting in the specific upregulation of the translation of activating transcription factor 4 (ATF4) and cardiomyocyte proliferation (Gao et al., 2023). Similarly, Nakada et al. gradually exposed mice to hypoxic conditions (7% O2) for 2 weeks and observed a reduction in mitochondrial oxygen radical production and the reactivation of myocardial cell mitosis(Figure 4) (Nakada et al., 2017).

The perinatal transformation in cardiomyocytes’ metabolism is characterized by an increase in blood oxygen saturation and a corresponding alteration in oxygen content, leading to the concept of reprogramming metabolism, particularly from the perspective of oxidized substrates, to mitigate the damage caused to genetic material by reactive oxygen species generated during mitochondrial oxygen metabolism (Table 3). Cardoso et al. have observed an increase in CM proliferation in neonatal mice fed with fatty acid-deficient milk, as well as an increase in cardiomyocyte proliferation in mice in which pyruvate dehydrogenase kinase 4 (PDK4) was specifically knocked out (Cardoso et al., 2020). Inactivation of carnitine palmitoyltransferase-1b to eliminate fatty acid oxidation in cardiomyocytes was found to reverse the maturation of cardiomyocyte genes, thereby enhancing their tolerance to hypoxia-induced injury and promoting regeneration after damage (Li X. et al., 2023). A recent study revealed extensive remodeling of sphingolipid metabolism during the regenerative process of neonatal hearts after injury. Ji et al. discovered that during regeneration, SphK1 and SphK2, isoenzymes that produce Sphingosine 1-phosphate (S1P), can differentially regulate myocardial proliferation (Figure 4) (Table 3) (Ji et al., 2024).

**TABLE 3:** Metabolic modulation that promotes cardiomyocyte proliferation.

Metabolic modulation	Mechanism	Function	Ref
Hypoxia	Inhibition of oxidative metabolism Decreased ROS production Cell cycle re-entry	Myocardial regeneration in adult mice and functional recovery following MI	Nakada et al. (2017)
Knockdown of mitochondrial protein translation	MSR ISR ATF4 activation	Increase in cytokinesis during cardiomyocyte proliferation Increase the proliferation of human induced pluripotent stem cell-derived CMs	Gao et al. (2023)
Feed neonates with fatty acid-deficient milk CM-specific PDK4 knockout	Inhibit fatty-acid utilization	Extended window of cardiomyocytes proliferation in neonates Promote CM proliferation in neonatal mice	Cardoso et al. (2020)
Inactivation of Cpt 1b	Accumulation of α-ketoglutarate Activation of KDM5 Demethylates broad H3K4me3 domains	Enhance tolerance to hypoxia-induced injury and regeneration after damage Promote CM proliferation in adult mice after MI	Li et al. (2020c)
Reactivation of SphK2	S1P-dependent histone acetylation	CM cell-cycle re-entry and cytokinesis Promote CM proliferation in adult mice after MI	Ji et al. (2024)
SphK1 depletion	limits autocrine activation of CFs by S1P	Restrict fibrotic scarring	

## 6 Cardiac dynamics

Heart rate, a biomarker of heart function, is the frequency of mechanical contraction of the heart. It is a determinant of myocardial oxygen consumption, coronary blood flow, and myocardial function. The autonomic nervous system can adjust the heart rate to modify cardiac output and adapt it to the metabolic requirements (Bohm and Reil, 2013). In certain diseases, heart rate can serve as a risk indicator and modifiable risk factor that can improve the outcome of cardiovascular disease. The continuous contraction of myocardial cells requires a large amount of ATP as an energy source, which may explain the unique energy metabolism and low regeneration ability of the myocardium (Lozhkin et al., 2022). According to a study by Tan et al., the heart rate of neonatal mice at 3 days is approximately 17% lower than that of 60-day-old mice. This study validated the essential role of reduced heart rate in myocardial regeneration across three injury models: neonatal rats, zebrafish with surgically induced heart injury, and adult mice with acute MI(Tan et al., 2022). Multiomic analysis showed that a reduced heart rate integrates two major biological features: metabolism patterns and proliferation capacity: (1) the reduced metabolic demand which shifts the primary energy source from fatty acid oxidation to glucose oxidation and catabolism; (2) the expression of key enzymes involved in glycolysis, such as PKM2, which is increased and plays a nonenzymatic role in promoting cell cycle progression; (3) the upregulation of cyclin D1 expression in cardiomyocytes which promotes the G1/S phase transition and leads to the reentry of cardiomyocytes into the cell cycle(Figure 3G) (Tan et al., 2022).

The morphological development of the heart is a highly intricate process, with mechanical forces generated by biological mechanisms playing a significant role. In the field of congenital heart disease research, compelling evidence suggests that abnormal biomechanics may contribute to the development of cardiac deformities. Shear and hydrostatic stress can regulate the tension of valvular ECs and the proliferation of valve interstitial cells through the YAP signaling pathway, thereby influencing the size and shape of the valve (Wang M. et al., 2023). Hoog et al. created low-hematocrit embryos by injecting acrylamide and TEMED (AT) into yolk sac blood island vessels and observed that reducing only the hemodynamic load had a significant impact on heart volume and myocardial thickness (Hoog et al., 2018). Mohammadi et al. established a model of cardiac pressure overload in neonatal mice via a transverse aortic constriction protocol and demonstrated that pressure overload can stimulate the proliferation of left ventricular cardiomyocytes and prevent adverse remodeling after injury (Malek Mohammadi et al., 2019). Consistent with previous findings, the experiments conducted by Ye et al. showed that pressure overload markedly enhances the proliferation of cardiomyocytes in both neonatal rats and humans (Ye et al., 2020).

In adult mammals, pressure overload frequently leads to a range of pathological alterations in the heart. Technological advancements are increasing the clinical application of left ventricular assist devices (LVADs) to improve cardiac output and organ function in patients with end-stage heart failure (Table 4) (Kirklin et al., 2014). In these patients a reduction in polyploid cardiomyocytes and a concurrent increase in diploid cardiomyocytes have been observed, suggesting that the restoration of myocardial regenerative capacity may be associated with the morphological restoration of the heart following pressure unloading (Wohlschlaeger et al., 2010). In agreement with previous studies, mechanical unloading has been shown to lead to cardiomyocyte cell cycle re-entry and heart regeneration; this highlights the need for a better understanding of the temporal effects of mechanical unloading on cardiomyocytes (Table 4) (Canseco et al., 2015).

**TABLE 4:** Cardiac dynamics that promote cardiomyocyte proliferation.

Cardiac dynamics modulation	Mechanism	Function	Ref
Moderate heart rate reduction	Shift the primary energy source from fatty acid oxidation to glucose oxidation PKM2 promote cell cycle progression Upregulate Cyclin D1 expression in CM	CM cell-cycle re-entry and cytokinesis Induce CM proliferation under physiological conditions Promote cardiac regenerative repair after MI Long-term CM restoration	Tan et al. (2022)
Pressure overload–associated maladaptation by TAC	Transcription factor GATA4	Induce CM proliferation and angiogenesis in neonatal mice with TAC	Malek Mohammadi et al. (2019)
Pressure overload (human samples and neonatal mice with PAB)	Unknown	Promote CMs proliferation in the right ventricle of neonatal mice (particular the 3-day postnatal mice) Increase of Ki67/pHH3/aurora B-positive CMs in human-overloaded right ventricles	Ye et al. (2020)
Pressure unloading (patients supported by LAD)	Cell cycle progression	Decrease in polyploidy and increase in diploidy Promote heart regeneration	Wohlschlaeger et al. (2010)
	Prevent mitochondria-mediated activation of DDR	Induce CM mitosis and cytokinesis Promote heart regeneration	Canseco et al. (2015)

## 7 Intracellular mechanism involved in heart regeneration

In general, injury triggers quiescent CMs to reenter the cell cycle through dedifferentiation, mitochondrial remodeling, and reduced adhesion (Zheng et al., 2021). This process typically occurs in a regenerative microenvironment. Transmission, regulation, and interaction of various molecular signals are important for the growth, repair, and regeneration of cardiomyocytes. The limited regenerative capacity of cardiomyocytes is a bottleneck that needs to be solved. To address this issue, it is necessary to deeply understand the intracellular molecular signals and pathways involved in the process of regeneration.

### 7.1 Cell cycle regulators

**FIGURE 5:**
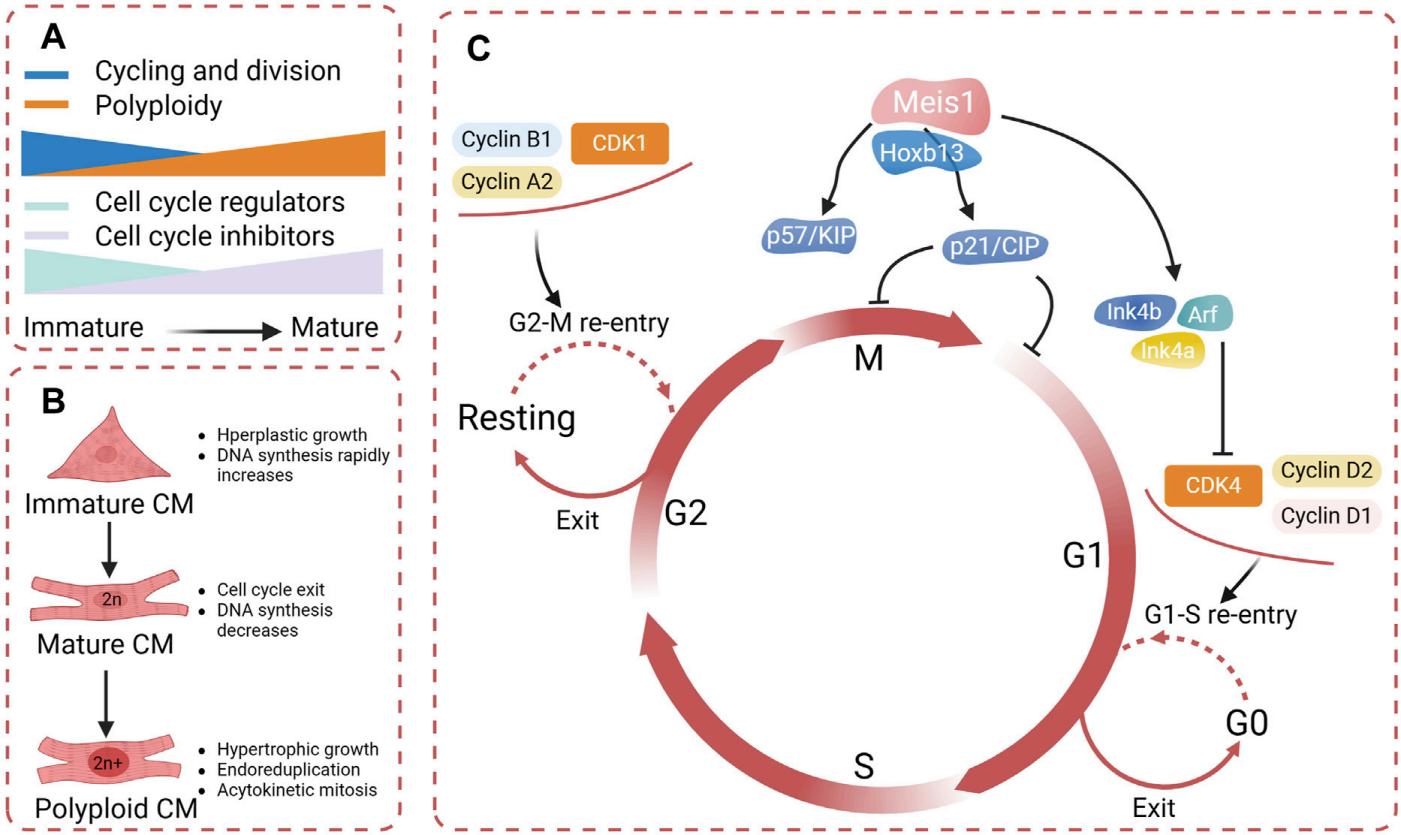
**(A)** During the process of cardiomyocyte differentiation and maturation, there is a decline in the expression of cell cycle regulators and a increase in cell cycle inhibitors, resulting in binucleation and the subsequent inability to proliferate further. **(B)** Cardiomyocytes undergo gradual binucleation and multinucleation during their maturation, resulting in acytokinetic mitosis. **(C)** Cell cycle regulators identified promote cardiomyocyte re-entry into the mitotic cycle (created with BioRender.com).

After birth, the expression of cell cycle regulators such as CDK2, CDK3, CDK4, CCND1, and CDK cofactors is downregulated, while the expression of cell cycle inhibitors CKIs (such as INK four and CIP/KIP family members) is upregulated, inducing cardiomyocytes to exit the cell cycle(Figure 5) (Uygur and Lee, 2016). During postnatal growth, cardiomyocytes gradually transition from hyperplastic growth to hypertrophic growth under various conditions. DNA synthesis rapidly increases and then gradually decreases to a very low level, and chromatin polyploidy increases (Velayutham et al., 2020). In the hypertrophic growth stage, cardiomyocytes develop cell cycle abnormalities, characterized by endoreduplication and acytokinetic mitosis, losing the ability to undergo nuclear and cytoplasmic division (Hesse et al., 2012). At 14 days after birth, most myocardial cells in the mouse ventricles become binuclear, 45% of which are polyploid (Walsh et al., 2010).

Exit from the cell cycle fundamentally limits the possibility of adult mammalian cardiomyocyte proliferation. Some research has focused on the cell cycle proteins to observe whether cardiomyocytes can reenter the cell cycle. The complex of the cell cycle regulator cyclin A2 and CDK1 promotes entry into mitosis and is essential for the G1/S transition (Mehdipour et al., 2023). Cyclin A2 is silenced after birth, which is consistent with the exit of cardiomyocytes from the cell cycle (Yoshizumi et al., 1995). Studies have convincingly shown that the induced expression of cyclin A2 in infarcted hearts can effectively facilitate myocardial regeneration and repair in both mice and porcine hearts (Chaudhry et al., 2004). Cyclin D1 and D2 have been shown to promote myocardial proliferation by activating CDK4 ligands (Eghbali et al., 2019). In 2018 Mohamed et al. utilized high-throughput screening to compare the expression of cell cycle regulatory genes in embryonic, neonatal, and adult mice and identified the most effective gene combination for CM proliferation: CDK1/CCNB/CDK4/CCND(Mohamed et al., 2018). After mitosis, the overexpression of these genes in mice and humans effectively stimulates CM proliferation and enhances repair postinfarction (Mohamed et al., 2018). Interestingly, in an experiment that regulated cardiomyocyte proliferation through cell cycle regulators, it was discovered that hypertrophy is correlated with cardiomyocyte proliferation. The cell cycle re-entry during hypertrophy can be converted through increased expression of activators such as cyclin D2 (Busk et al., 2005). This further suggests that by precisely modulating cell cycle regulators to induce cardiomyocytes into a mitotic state, we can not only effectively restore the loss of functional cardiomyocytes, but also can inhibit pathological myocardial remodeling (Gong et al., 2021).

Meis1, a representative of the Meis homeobox genes, is a transcription factor that plays an essential role in heart development and regulates postnatal cell cycle arrest in cardiomyocytes (Mahmoud et al., 2013). In mice, deletion of Meis1 extends the postnatal window, enabling adult cardiomyocytes to resume the cell cycle (Mahmoud et al., 2013). Evidence suggests an interaction between Meis1 and the Ink4b–Arf–Ink4a and p21 promoters in adult mice (Mahmoud et al., 2013). Meis1 deficiency leads to the downregulation of cyclin-dependent kinase inhibitors in cardiomyocytes, specifically those encoded by the Ink4b–Arf–Ink4a locus (p16, p15, and p19Arf) and the CIP/KIP family (p21 and p57) (Mahmoud et al., 2013). A decrease in Meis1 expression has been associated with the shift from glycolysis to oxidative metabolism following birth, which is considered a hallmark of cellular maturity (Li Y. et al., 2022). Hoxb13, a member of the homeobox gene family, plays a key role in the regulation of cell growth and differentiation (Li M. et al., 2023). Nguyen et al. demonstrated that Hoxb13 serves as a cofactor for Meis1 in postnatal cardiomyocytes to regulate their maturation and proliferation (Nguyen et al., 2020). Meis1 also interacts with other genes and signaling pathways to jointly participate in the fate determination and functional regulation of cardiomyocytes. Along with most of the Hoxa genes, Meis1 is downregulated during monocyte-macrophage terminal differentiation, which is reparative and causes minimal inflammation (Paul et al., 2020). In summary, to develop a treatment for myocardial regeneration by manipulating cell cycle proteins, a thorough understanding of their specific functions in cardiomyocyte growth and proliferation, as well as their interactions with other signaling molecules, is essential (Figure 5).

Although studies have shown that activation of the cell cycle can induce proliferation of cardiomyocytes. In the context of heart disease and aging, it is noteworthy that cell cycle dysregulation in cardiomyocytes has been intricately linked to the development of cardiac pathologies (Lee et al., 2009). The activation of the cell cycle also exerts significant effects on replication stress and DNA damage in other research (Nixon et al., 2017; Pal et al., 2021). This is particularly problematic in the aging heart, where cardiomyocytes become less efficient at repairing DNA damage and responding to stress (Choudhury et al., 2022). In summary, while activation of the cell cycle has the potential to induce cardiomyocyte proliferation and improve heart function, it also poses significant risks in terms of increased replication stress and DNA damage.

### 7.2 The Hippo-YAP/TAZ pathway

**FIGURE 6:**
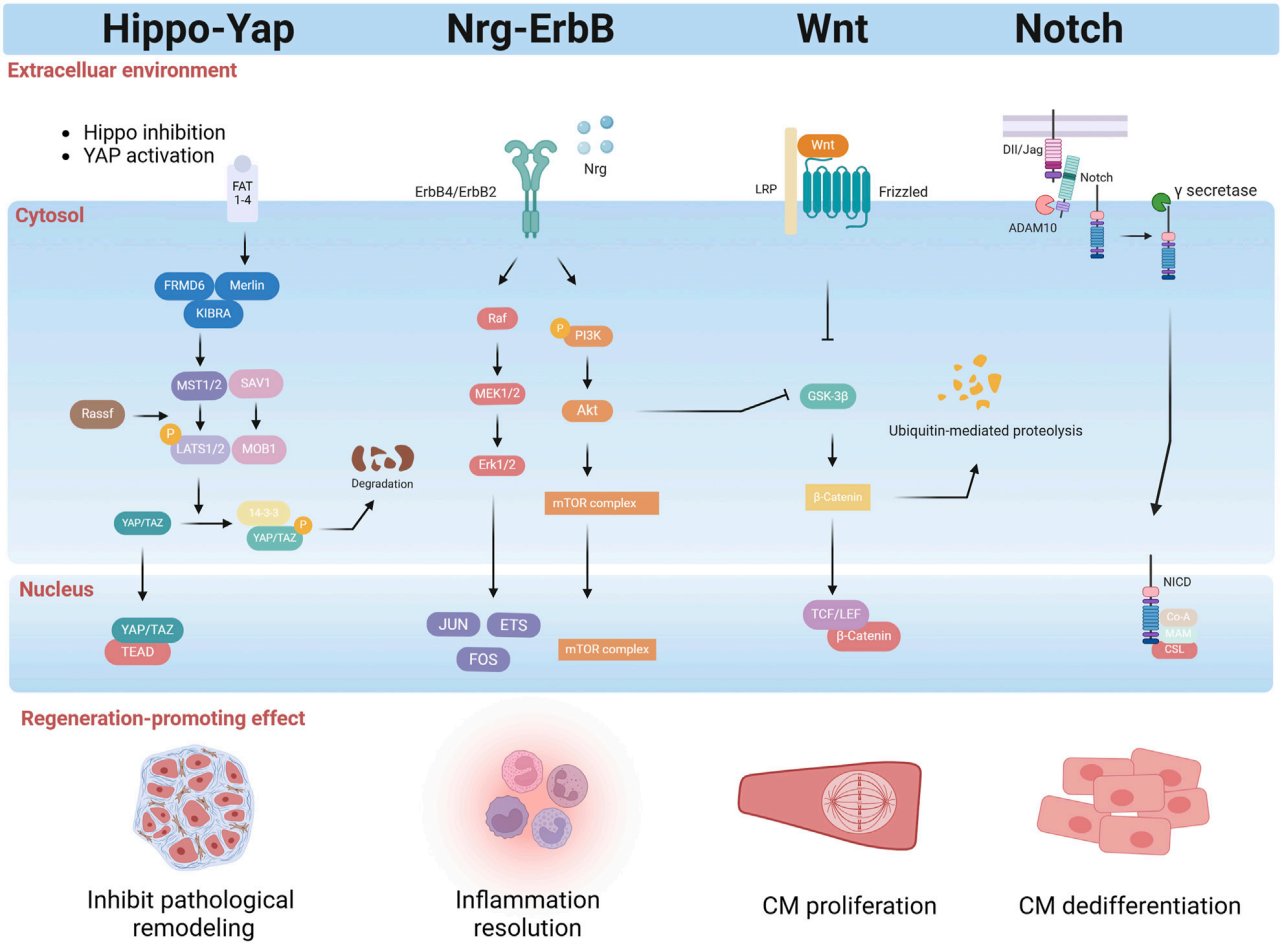
Regulatory factors released by the myocardial microenvironment effectively promote cardiomyocyte regeneration by activating intrinsic mechanisms within the cells. These factors not only stimulate cardiomyocyte proliferation and differentiation but also initiate tissue repair, accelerating the recovery of damaged cardiomyocyte. Furthermore, they modulate inflammatory responses, mitigating inflammatory damage, and suppress pathological remodeling, reducing myocardial fibrosis and scarring, thus preserving cardiomyocyte structure and function. Collectively, these regulatory factors play a crucial role in cardiomyocyte proliferation and heart regeneration (created with BioRender.com).

The Hippo pathway is a highly conserved pathway that regulates cell proliferation, differentiation, and survival and influences organ size. In mammals, the Hippo pathway consists of a serine/threonine kinase phosphorylation cascade, which includes STE20-like kinases one and 2 (MST1/2), large tumor suppressor homologs one and 2 (LATS1/2), and two downstream transcriptional coactivators: Yes-associated protein (YAP) and PDZ-binding motif (TAZ) (Figure 6) (Xie et al., 2022). In the heart, Hippo pathway activity increases with age, while YAP activity gradually decreases after birth, contributing to the loss of cardiac regenerative capacity after birth (Heallen et al., 2013). The Hippo pathway is highly conserved in mammals and senses extracellular signals related to mechanical force, cell polarity, cell adhesion, and nutrient availability (Riley et al., 2022). Nonphosphorylated YAP/TAZ can enter the nucleus, where it can bind with transcription factors such as TEAD domain-containing proteins to regulate downstream genes (Wang et al., 2018). Once LATS1/2 senses inhibitory signals in the environment, it promotes the phosphorylation of YAP/TAZ, which remains in the cytoplasm and is degraded, thereby preventing its translocation to the nucleus and inducing the expression of genes involved in proliferation and organ size (Liu et al., 2022). The results of the present study indicated that the Hippo–YAP/TAZ signaling pathway comprehensively regulates heart regeneration by directly regulating the endogenous myocardial proliferative capacity and coordinating intercellular communication in cardiomyocytes (Liu et al., 2022).

Leach et al. analyzed human ischemic and nonischemic heart failure samples and showed that, compared to control donor samples, there was an increase in Hippo signaling pathway activity (Flinn et al., 2020). In adult mice with ischemic heart failure induced by MI, knockout of tumor suppressor Sav1 in cardiomyocytes can reverse systolic HF; cardiomyocyte regeneration has been observed in mice after administration with AAV9 expressing shRNA targeting Sav1 (Leach et al., 2017). Monroe et al. partially reprogrammed adult mice, using an active version of the Hippo pathway effector YAP5SA, and found that YAP5SA could transform highly differentiated cardiomyocytes into a primitive, fetal-like, proliferative state *in vivo* (Monroe et al., 2019). Tian et al. discovered that the miR-302–367 microRNA cluster plays a pivotal role in CM proliferation during development, as its overexpression can reactivate the cell cycle by suppressing the Hippo pathway (Tian et al., 2015).

The Hippo-YAP signaling pathway not only coordinates noncardiomyocyte environmental signals but also plays a crucial role in cardiomyocyte regeneration. The immune system’s interaction with the Hippo signaling pathway is vital for cardiac regeneration. Mia et al. reported that YAP and TAZ can promote IL-6 expression while simultaneously reducing Arg1 expression by interacting with the histone deacetylase 3 (HDAC3)-nuclear receptor corepressor 1 (NCoR1) repressor complex, which leads to the promotion of a proinflammatory phenotype and the inhibition of a reparative macrophage phenotype (Mia et al., 2020). This finding suggested that the Hippo-YAP/TAZ pathway may serve as an important regulator in the immune-macrophage-mediated regenerative repair response following MI. The Hippo-YAP pathway can also participate in crosstalk with the NRG/ErbB2 signaling pathway during cardiac regeneration. Overexpression of ErbB2 in cardiomyocytes leads to the phosphorylation of YAP at S352 and S274, activating YAP and promoting an epithelial-mesenchymal transition-like regenerative response in a heart failure model (Aharonov et al., 2020). A study also suggested that ERBB4 activated by neuregulin one can serve as a “dedicated” receptor for the Hippo-YAP signaling pathway, but this pathway has not yet been studied in the context of cardiomyocyte regeneration (Sudol, 2014). Other studies show that the Hippo/YAP signaling pathway interacts with the β-catenin pathway and the PI3K/Akt pathway, regulating cardiomyocyte regeneration (Zheng et al., 2021). In summary, the Hippo-YAP signaling pathway plays a central role in mammalian heart regeneration and repair. It not only directly regulates cardiomyocyte proliferation and differentiation, but also coordinates with noncardiomyocyte environmental signals to promote cardiomyocyte regeneration.

### 7.3 The NRG1/ErbB signaling pathway

The NRG1/ErbB signaling pathway plays a crucial role in heart development by regulating CM proliferation, differentiation, maturation, and morphology. Neuregulin1 (NRG1), a member of the epidermal growth factor receptor family, is an agonist of receptor tyrosine kinases which, when bound to ErbB4, can increase its kinase activity and lead to heterodimerization with ErbB2 or homodimerization with ErbB4, ultimately resulting in the stimulation of intracellular signaling pathways(Figure 6) (Wang B. et al., 2023). Bersell et al. (2009) demonstrated that adult cardiomyocytes can be induced to proliferate by activating the NRG1/ErbB4 signaling pathway, thereby enhancing regeneration and repair following MI(Bersell et al., 2009). Polizzotti et al. (2015) evaluated the effectiveness of recombinant growth factor neuregulin-1 (rNRG1) administration in cardiac regeneration and found that rNRG1 is crucial for cardiomyocyte regeneration in a murine model of P1 cryoinjury; they further found that rNRG1 stimulated proliferation of cultured cardiomyocytes from pediatric patients less than 6 months of age (Polizzotti et al., 2015). Similarly, genetic activation of ERBB2 after MI triggered CM dedifferentiation and proliferation followed by redifferentiation and regeneration (DUva et al., 2015). However, several studies have failed to demonstrate that rNRG1 enhances cardiomyocyte renewal rates in adult mice, either under normal circumstances or following MI(Reuter et al., 2014). In a clinical trial, 40 patients with significant left ventricular dysfunction and heart failure showed a dose-dependent improvement in the left ventricular ejection fraction after 90 days of treatment with cimaglermin Alfa (Neuregulin 1b3) (Lenihan et al., 2016).

Other studies have also shown that the NRG1/ErbB signaling pathway interacts with other pathways to promote cardiomyocyte regeneration. Recently, Honkoop et al. utilized single-cell sequencing and discovered that activation of the NRG1/ErbB2 signaling pathway can induce regeneration by metabolically reprogramming border zone CMs, switching their energy production from oxidative phosphorylation to glycolysis (Honkoop et al., 2019). Aharonov et al. showed that ERBB2–ERK–YAP mechanotransduction signaling regulates cardiac regeneration (Aharonov et al., 2020). In summary, while the NRG1/ErbB signaling pathway has gained significant attention for its role in myocardial regeneration and repair, further research is required to fully comprehend its specific function in the adult heart.

### 7.4 Notch signaling pathway

The Notch signaling pathway is a highly conserved signaling cascade that plays a critical role in regulating tissue development, differentiation, and regeneration. Before engaging in ligand-receptor interactions at the cell surface, both Notch receptors and ligands undergo complex routing and modification (Li et al., 2021). The Notch receptor is a single-pass transmembrane protein consisting of epidermal growth factor (EGF)-like repeats and a negative regulatory region (NRR) (Siebel and Lendahl, 2017). In mammals, Notch signaling pathway consists of four components: the Notch receptors (Notch1/2/3/4), the Notch ligands (Delta-like ligands, DLL1/3/4 and Jagged ligands, JAG1/2), CSL-DNA binding proteins, and downstream target genes(Figure 6) (Siebel and Lendahl, 2017). Raya et al. first reported the upregulated expression of the Notch receptor and ligand in the regeneration of amputated adult zebrafish hearts in 2003 (Raya et al., 2003). In the zebrafish apical amputation model, Notch is activated following injury in the epicardium and endocardium, promoting cardiomyocyte regeneration. However, excessive activation of the Notch signaling pathway is incompatible with myocardial proliferation, calling attention to the crosstalk between Notch and other signaling hubs during regeneration (Zhao et al., 2014). Similarly, activation of Notch1 or its ligand Jagged1 by adeno-associated virus gene transfer increases the proliferative capacity of neonatal cardiomyocytes (Felician et al., 2014).

A recent study explored the role of the Notch signaling pathway in myocardial regeneration. The Notch signaling pathway is a complex network that interacts with other signaling pathways to coordinately regulate CM proliferation and regeneration. Zhao et al. (2019) demonstrated that Notch signaling supports CM proliferation by dampening myocardial Wnt activity during zebrafish heart regeneration (Zhao et al., 2019). In mammals, the upregulation of Notch signaling during the early postnatal period can reactivate the cell cycle of quiescent cardiomyocytes by inducing the expression and nuclear localization of Cyclin D1, allowing them to reenter the cell cycle and proliferate (Siebel and Lendahl, 2017). A study by Li et al. demonstrated that the primary cilia of the endocardium can upregulate the expression of the Klf2 gene in response to hemodynamic alterations, thereby activating the Notch signaling pathway and promoting heart regeneration (Li X. et al., 2020). The levels of DLL1 (a Notch ligand), Notch1 (a Notch receptor), and connective tissue growth factor amphiregulin (Areg) (YAP targets) were all significantly elevated in cardiomyocytes undergoing OSM-induced proliferation (Li Y. et al., 2020).

### 7.5 Wnt signaling pathway

The Wnt signaling pathway, an evolutionarily conserved mechanism, assumes contradictory roles in the various stages of embryonic heart development, ultimately influencing cardiomyocyte specification and differentiation in a pivotal manner (Buijtendijk et al., 2020). It is also essential for the regulation of cardiac homeostasis, fibrosis, injury repair, and regeneration (Li D. et al., 2022). Three Wnt signaling pathways have been characterized: the canonical Wnt pathway (Wnt/β-catenin pathway), the noncanonical Wnt/planar cell polarity pathway (Wnt/PCP pathway), and the noncanonical Wnt/Ca^2+^ pathway (Wnt/Ca^2+^ pathway) (Figure 6). These three pathways are all regulated by the binding of Wnt protein ligands to the seven-transmembrane protein family of receptors (frizzled, Fzd) on the cell surface (Cailotto and Santulli, 2023). Although quiescent in the adult mammalian heart, the Wnt pathway is activated upon injury and plays a significant role in cardiac repair (Li Y. et al., 2022). Bastakoty and colleagues reported that temporary, systemic inhibition of the Wnt/β-Catenin pathway immediately following MI increased proliferation of cardiac progenitors and reduced adverse remodeling infarct size in mice (Bastakoty et al., 2016). A recent study showed that the Wnt signaling inhibitor CGX1321 can stimulate the proliferation of cardiomyocytes following MI by regulating genes related to the cell cycle (Shah et al., 2021). Consistent with these findings, specific deletion of LRP6, a coreceptor of Wnt ligands, in mice cardiomyocytes, increased cardiomyocyte cell cycle activity in adult mice and induced a robust regenerative response after MI (Wu et al., 2021). Cardiomogen (CDMG), a novel Wnt inhibitor, was identified in mice following LAD ligation to enhance cardiomyocyte regeneration in the infarct border zone (Xie et al., 2020). Mechanistically, Wnt signaling inhibition and PAK2-mediated pS675-b-catenin signaling function coordinately to promote CM dedifferentiation and proliferation in zebrafish heart regeneration (Peng et al., 2021). In injured zebrafish hearts, however, the expression of Wnt ligands (including Wnt4a, Wnt6b, and Wnt8a) is reduced, while the expression of noncanonical Wnt2bb is increased (Peng et al., 2020). Another study reported that Wnt/β-catenin has opposing roles in the nonregenerating adult rodent heart, where it appears to inhibit repair, and in the regenerating zebrafish heart, where it seems to promote regeneration. These findings indicate that Wnt activation and inhibition are needed for heart regeneration at different stages, like the biphasic Wnt signaling during heart development (Li Y. et al., 2022).

## 8 Conclusion and perspectives

Overall, the profound research in this field has revealed the molecular mechanisms and potential modulators of heart regeneration. To precisely investigate human cardiovascular diseases, researchers have successfully established various animal models to mimic human pathologies. These models have provided significant insights into myocardial regeneration, yet they fall short in capturing the pathophysiological complexity and molecular mechanism diversity of cardiovascular diseases. Consequently, there is a need for more efficient research approaches that integrate advanced techniques such as genomics, proteomics, metabolomics, and epigenetics to unravel the intricate networks and interactions involved in the progression of cardiovascular diseases. Furthermore, a thorough analysis of clinical data from patients with heart disease is crucial, as it can provide valuable insights into the regenerative outcomes.

Cardiomyocytes have a limited capacity for regeneration, which is the reason that heart damage caused by infarction or other insults often leads to permanent loss of muscle tissue. Recent research has highlighted the role of noncardiomyocytes in cardiac regeneration. These cells, including cardiac fibroblasts, endothelial cells, and immune cells, play a crucial role in tissue repair and regeneration (Table 1). Although cardiomyocytes have traditionally been considered the primary players in myocardial regeneration, the role of noncardiomyocytes is increasingly recognized. Understanding the interactions between these cells and cardiomyocytes holds promise for developing new therapeutic strategies aimed at enhancing myocardial regeneration following injury. Future research should focus on deciphering the complex network of signaling pathways and mediators involved in cardiac regeneration and on identifying novel targets for therapeutic intervention.

In the process of cardiomyocyte regeneration, if we compare the regenerative cardiomyocyte to “germination,” the cardiomyocytes act as the “seed.” To support their growth, a more “fertile soil” or favorable modulator of the myocardial environment is required to promote regeneration. This “soil” consists of various cell types and factors that provide the necessary nutrients, signals, and microenvironment for cardiomyocytes to thrive and multiply. The interaction between cardiomyocytes and this supportive cellular environment is crucial for myocardial regeneration and repair following injury. Future research should aim to identify the key components of this regenerative “soil” and determine how to optimize it to enhance myocardial regeneration *in vivo*.

## Abbreviations

MI, myocardial infraction; CM, cardiomyocyte; MHC, major histocompatibility complex; USSC, somatic Stem Cells; EC, endothelial cell; LEC, lymphatic Endothelial cell; ATF4, activating transcription factor 4; PDK4, pyruvate dehydrogenase kinase 4; S1P, sphingosine 1-phosphate; TNF-α, tumor necrosis factor α; TGF-β, transforming growth factor β; STAT3, signal transducer and activator of transcription 3; IFN-γ, interferon-gamma; MSR, mitochondrial stress response; ISR, integrated stress response; LVAD, left ventricular assist device; NRR, negative regulatory region.
